# Long‐Term Survival Analysis of Neoadjuvant Chemoradiotherapy Versus Adjuvant Chemoradiotherapy for Locally Advanced Low Rectal Cancer

**DOI:** 10.1002/cam4.71042

**Published:** 2025-07-25

**Authors:** Siyuan Chen, Ruiyan Wu, Juefeng Wan, Yun Xu, Yaqi Wang, Zhiyuan Zhang, Lili Huang, Yujun Liu, Yingxuan Lin, Luoxi He, Yun Deng, Fan Xia, Ye Xu, Zhen Zhang, Hongtu Zheng

**Affiliations:** ^1^ Department of Radiation Oncology Fudan University Shanghai Cancer Center Shanghai China; ^2^ Department of Oncology Shanghai Medical College, Fudan University Shanghai China; ^3^ Shanghai Clinical Research Center for Radiation Oncology Shanghai China; ^4^ Shanghai Key Laboratory of Radiation Oncology Shanghai China; ^5^ Department of Colorectal Surgery Fudan University Shanghai Cancer Center Shanghai China

**Keywords:** adjuvant chemoradiotherapy, locally advanced low rectal cancer, neoadjuvant chemoradiotherapy, oncologic outcome, overall survival

## Abstract

**Purpose:**

To compare the long‐term survival of patients with locally advanced low rectal cancer (LALRC), receiving neoadjuvant chemoradiotherapy (NCRT) versus adjuvant chemoradiotherapy (ACRT).

**Methods and Materials:**

This retrospective observational study included 1169 patients with LALRC (Stage II/III disease located ≤ 5 cm from the anal verge) who underwent diagnosis and treatment at Fudan University Shanghai Cancer Center from February 2006 to March 2021. In Stage II/III low rectal cancer patients, one‐to‐one matched pairs were created from the ACRT and NCRT groups using propensity score matching (PSM) based on baseline characteristics. OS and DFS were evaluated using the Kaplan–Meier method alongside the univariate Cox regression model.

**Results:**

In Stage II patients, 65 received ACRT and 107 received NCRT. For Stage III, 282 received ACRT and 715 received NCRT. After PSM, 45 paired Stage II patients and 243 paired Stage III patients were selected. In Stage II patients, there was no significant difference in OS and DFS between the groups. For Stage III, the 5‐ and 10‐year OS rates were 79.61% and 77.67% in the NCRT group, compared to 61.08% and 44.57% in the ACRT group (*p* < 0.001). The 5‐ and 10‐year DFS rates were 69.93% and 65.26% in the NCRT group, versus 48.07% and 40.77% in the ACRT group (*p* < 0.001). Additionally, in Stage III patients, NCRT was associated with a significant reduction in the risk of death and recurrence compared to ACRT (OS: HR = 0.47, *p* = 0.0001; DFS: HR = 0.55, *p* = 0.0001).

**Conclusion:**

For patients with Stage III low rectal cancer, NCRT significantly improved the long‐term DFS rate and OS rate, in comparison to adjuvant chemoradiotherapy.

## 1 Introduction

In 2020, colorectal cancer ranked as the third most frequently diagnosed cancer worldwide and was the second leading cause of cancer‐related mortality [1]. It was estimated that the global incidence of new colorectal cancer cases exceeded 1.9 million, with deaths reaching approximately 935,000 [1]. Approximately 25%–50% of colorectal cancer cases are rectal cancer [2, 3], and around 15% of colorectal cancer patients are diagnosed with locally advanced rectal cancer (LARC) at diagnosis [4]. LARC is characterized by significant invasion beyond the muscular layers of the rectal wall or involvement of lymph nodes within the mesorectum and true pelvis (c/pT3‐4 or c/pN+), without distant metastasis [5, 6].

Patients with LARC are at an increased risk of local recurrence and distant metastasis [7, 8, 9], posing a challenge for comprehensive treatment. The implementation of Total Mesorectal Excision (TME) has led to a substantial reduction in local recurrence rates when compared to conventional surgical techniques, but the local recurrence rate of patients with LARC still remains as high as 30% [10]. In the 1990s, multiple clinical trials demonstrated that postoperative chemoradiotherapy (adjuvant chemoradiotherapy, abbreviated as ACRT) could significantly lower recurrence rates and enhance overall survival relative to surgery alone or surgery combined with irradiation, thereby confirming its importance in treating patients with LARC [11, 12, 13].

Subsequently, preoperative chemoradiotherapy (neoadjuvant chemoradiotherapy, abbreviated as NCRT), has also been widely adopted for the treatment of patients with LARC. NSABP R‐03, CAO/ARO/AIO‐94, and MRC CR07, these notable phase III randomized clinical trials have shown that NCRT is superior to ACRT in terms of inducing tumor regression, reducing local recurrence, ameliorating the toxicity and increasing the rate of sphincter preservation [13, 14, 15, 16]. It also offers the potential for a complete clinical response (cCR) and the application of a ‘watch and wait’ approach [17, 18], which can spare patients from surgery. Moreover, patients who achieve a pathological complete response (pCR) after NCRT have a 5‐year overall survival rate of nearly 90% [14]. The integration of NCRT and TME is now a standard treatment modality for LARC, as recommended by the National Comprehensive Cancer Network (NCCN) guidelines [19].

Despite these advancements, controversy persists in clinical practice regarding whether NCRT improves patient survival compared to ACRT. Although NSABP R‐03 and MRC CR07 found NCRT could improve disease‐free survival (DFS) compared to ACRT, all three phase III randomized clinical trials mentioned above did not find significant improvement in overall survival (OS) [13, 14, 15, 16].

Furthermore, in patients with T3N0/*N*+ or T2N+ who have a free margin of at least 2 mm from the mesorectal fascia, TME alone can achieve a local recurrence rate below 6% [20]. As a result, many surgeons argue that for these patients, employing only TME is sufficient to achieve satisfactory oncologic outcomes, and given that NCRT fails to improve the overall survival of these patients, it is suggested that patients forego NCRT to avoid overtreatment, except when sphincter preservation or avoidance of surgery is the goal.

What's more, the PROSPECT trial rendered an argument that the neoadjuvant chemotherapy was noninferior to neoadjuvant chemoradiotherapy regarding the disease‐free survival [21], providing evidence for omission of radiotherapy in the operative treatment of locally advanced cancer.

However, previous studies on the impact of NCRT on survival have often analyzed patients with LARC as a whole, lacking more detailed stratification. In our research, we focus on patients with locally advanced low rectal cancer (LALRC, the distance of tumor from anal verge ≤ 5 cm), who often receive NCRT in order to achieve sphincter preservation and even surgery avoidance. We retrospectively evaluate the effect of NCRT on long‐term oncologic outcomes in patients with LALRC. Despite the findings of the PROSPECT trial, our data suggest that we be cautious with the omission of radiotherapy, because if patients ultimately need ACRT, their overall survival might be undermined.

## 2 Methods and Materials

### 2.1 Eligibility

A total of 1169 patients with LALRC, who underwent diagnosis and treatment at Fudan University Shanghai Cancer Center from February 2006 to March 2021, were retrospectively analyzed.

All included patients satisfied the following criteria: (1) primary locally advanced rectal cancer (T3/4 or N+, M0) confirmed by MRI; (2) pathologically diagnosed with rectal adenocarcinoma by colonoscopy biopsy; (3) the distance of the tumor from the anal verge was ≤ 5 cm at the first diagnosis; (4) equipped with complete clinical data and follow‐up information.

This study adheres to the American Joint Committee on Cancer (AJCC) 8th edition staging criteria; detailed diagnostic standards can be found within the 8th edition staging guidelines [22].

Patients are excluded with the following conditions: (1) existence of distant metastasis; (2) presence of tumor in other sites; (3) patients with a history of tumor.

Written, informed consent for the therapeutic procedures was obtained from all patients.

### 2.2 Treatment Protocols

For NCRT, patients received pelvic long‐course radiotherapy with a planned dose of 50 Gy delivered in 25 fractions over 5–6 weeks with concurrent administration of capecitabine(825 mg/m^2^ twice daily for 5 days/week). Capecitabine plus oxaliplatin (CapeOX, capecitabine 1000 mg/m^2^ twice daily on Day 1–14 and oxaliplatin 130 mg/m^2^ on Day 1) was administered as consolidation chemotherapy for at least one cycle. The exact number of cycles of consolidation chemotherapy was based on the interval between radiotherapy and radical surgery. The decision to prescribe adjuvant chemotherapy was left to the discretion of the treating physician, and, in general, the combined number of consolidation and adjuvant chemotherapy cycles totaled six. For ACRT, the planned dose for pelvic long‐course radiotherapy was set at 45 Gy, with the tumor bed being boosted to 50 Gy, administered in 25 fractions over 5–6 weeks, alongside concurrent administration of fluorouracil or capecitabine. Adjuvant chemotherapy using the CapeOX regimen was split between the pre‐ and post‐ radiotherapy periods, with a cumulative total of six cycles.

Radical resection of rectal cancer is given, including Dixon's operation, Miles' operation, and Hartmann's operation. The surgery follows the principle of TME, encompassing both laparoscopic surgery and open surgery. The assessment of resection completeness was performed based on the residual tumor classification.

### 2.3 Follow‐Up

The follow‐up protocol for patients who had TME adhered to the guidelines set forth by the NCCN. Clinical study endpoints were OS, DFS, local‐regional recurrence‐free survival (LRFS), and distant metastasis‐free survival. Patients were followed up until the cut‐off date (September 22, 2023), or until the event of death or tumor recurrence occurred.

### 2.4 Statistical Analysis

R, version 4.3.2 was used to perform statistical analyses. OS, DFS, LRFS, and DMFS were calculated using Kaplan–Meier curves. OS was measured from the date of surgery to the date of the last follow‐up or death. DFS was measured from the date of surgery to the date of tumor recurrence, the last follow‐up, or death. LRFS was measured from the date of surgery to the date of localregional tumor recurrence, the last follow‐up, or death. DMFS was measured from the date of surgery to the date of distant metastasis, the last follow‐up, or death. Continuous variables were analyzed using Student's *t*‐test and the Mann–Whitney *U*‐test. Categorical variables were analyzed using the chi‐square test and Fisher's exact test. Survival curves were compared using the log‐rank test. Differences were considered significant if the two‐sided *p*‐value was less than 0.05.

### 2.5 Propensity Score Matching (PSM)

To mitigate the possible confounding influences of therapeutic interventions and selection discrepancies, PSM was utilized to create a quasi‐randomized experimental framework. Following this, patients in clinical Stages II and III were matched in a one‐to‐one ratio between the NCRT and ACRT groups using the “matching” package in R to optimize the propensity score match. The propensity scores were estimated using a parsimonious logistic regression model with baseline variables: the distance of tumor from the anal verge, gender, age, clinical T stage, clinical N stage, clinical extramural vascular invasion (cEMVI) status, clinical mesorectal fascia (cMRF) status, smoking, alcoholism, hypertension, and surgery type. The matching was conducted using a nearest‐neighbor algorithm without replacement, with the caliper set to 0.20.

## 3 Results

### 3.1 Patient Characteristics

Our study enrolled 1169 patients with LALRC, with 822 treated with NCRT (NCRT group) and 347 treated with ACRT (ACRT group). Table 1 lists the patient, tumor, and treatment characteristics of both groups. Between the two groups, clinical staging, gender, pEMVI, pPNI, and surgery type were significantly different, while other factors did not distribute differently between the two groups. In the NCRT group, clinical Stage III tumors accounted for 86.98%, males for 70.92%, and 64.48% of patients underwent Miles' surgery. In contrast, the ACRT group had 81.27% clinical Stage III tumors, 56.77% males, and 72.05% underwent Miles' surgery (*p* < 0.05, Table 1).

**TABLE 1:** Characteristics of LALRC patients.

Characteristic^b^	ACRT (*n* = 347)	NCRT (*n* = 822)	*p* ^a^
Gender, *n* (%)			
Male	150 (43.2)	239 (29.1)	< 0.0001
Female	197 (56.8)	583 (70.9)	
Age (years), median [IQR]	56.0 [47.0, 63.0]	56.0 [48.0, 63.0]	0.8918
Smoking, *n* (%)			
No	332 (95.7)	769 (93.6)	0.2001
Yes	15 (4.3)	53 (6.4)	
Hypertension, *n* (%)			
No	326 (93.9)	767 (93.3)	0.7832
Yes	21 (6.1)	55 (6.7)	
Alcoholism, *n* (%)			
No	336 (96.8)	796 (96.8)	1
Yes	11 (3.2)	26 (3.2)	
Distance from anal verge (cm), median [IQR]	4.0 [3.0, 5.0]	4.0 [3.0, 5.0]	0.3818
Clinical staging, *n* (%)			
II	65 (18.7)	107 (13.0)	0.0151
III	282 (81.3)	715 (87.0)	
cEMVI, *n* (%)			
Negative	194 (55.9)	501 (60.9)	0.1088
Positive	153 (44.1)	321 (39.1)	
cMRF, *n* (%)			
Negative	243 (61.4)	529 (64.4)	0.9729
Positive	134 (38.6)	293 (35.6)	
Radiotherapy‐to‐surgery interval (weeks), median [IQR]	NA	8.6 [7.3,10.9]	NA
Surgery type, *n* (%)			
Dixon	86 (24.8)	250 (30.4)	0.0332
Miles	250 (72.0)	530 (64.5)	
Hartmann	11 (3.2)	42 (5.1)	
pEMVI, *n* (%)			
Negative	198 (57.1)	717 (87.2)	< 0.0001
Positive	143 (41.2)	60 (7.3)	
Unknown	6 (1.7)	45 (5.5)	
pPNI, *n* (%)			
Negative	213 (61.4)	678 (82.5)	< 0.0001
Positive	130 (37.5)	101 (12.3)	
Unknown	4 (1.1)	43 (5.2)	
CRM, *n* (%)			
Negative	332 (95.7)	803 (97.7)	0.0931
Positive	15 (4.3)	19 (2.3)	
Surgery‐to‐radiotherapy interval (weeks), median [IQR]	11.9 [9.9, 14.9]	NA	NA
Adjuvant chemotherapy			
No	0 (0.0)	76 (9.2)	NA
Yes	347 (100.0)	746 (90.8)	

Abbreviations: ACRT, adjuvant chemoradiotherapy; cEMVI, clinical extramural vascular invasion; cMRF, clinical mesorectal fascia; CRM, circumferential resection margin; LALRC, locally advanced low rectal cancer; NCRT, neoadjuvant chemoradiotherapy; pEMVI, pathological EMVI; pPNI, pathological perineural invasion.

^a^ The *p*‐values indicate the statistical significance of differences in variable distributions between the NCRT group and ACRT group. Continuous variables were analyzed using Student's *t*‐test (normally distributed data) or the Mann–Whitney *U*‐test (non‐parametric data). Categorical variables were compared with the chi‐square test or Fisher's exact test as appropriate. All tests were two‐sided, and a *p* < 0.05 was considered statistically significant.

^b^ Regarding pEMVI, pPNI, and CRM, these variables were determined based on postoperative pathology. At that time, for patients who underwent NCRT, they had received chemoradiation before surgery, while patients with ACRT did not receive any chemoradiation before surgery.

Given the significant impact of staging on long‐term survival, we categorized patients into Stage II (patients with clinical Stage II tumors) and Stage III (patients with clinical Stage III tumors) and described their baseline characteristics separately. In Stage II, 107 patients received NCRT, while 65 received ACRT. Table 2 listed the characteristics for Stage II, with significant differences in clinical/pathological T stage and age between the two groups. In Stage III, 715 patients were treated with NCRT, while 282 received ACRT. Table 3 detailed the characteristics for Stage III, with significant differences in clinical/pathological T stage, clinical/pathological N stage, clinical EMVI status, gender, and type of surgery between the two groups.

**TABLE 2:** Characteristic factors of Stage II patients before and after propensity matching.

Characteristic^b^	Before propensity matching			After propensity matching		
	ACRT (*n* = 65)	NCRT (*n* = 107)	*p* ^*a*^	ACRT (*n* = 45)	NCRT (*n* = 45)	*p* ^*a*^
Gender, *n* (%)						
Male	41 (63.0)	82 (76.6)	0.0826	13 (28.9)	13 (28.9)	1
Female	24 (36.9)	25 (23.4)		32 (71.1)	32 (71.1)	
Age (year), mean (SD)	59.7 (12.3)	55.2 (10.6)	0.0122	56.9 (12.7)	56.1 (9.1)	0.7253
Smoking, *n* (%)						
No	59 (90.8)	95 (88.8)	0.8766	40 (88.9)	38 (84.4)	0.7565
Yes	6 (9.2)	12 (11.2)		5 (11.1)	7 (15.6)	
Hypertension, *n* (%)						
No	62 (95.4)	101 (94.4)	1	44 (97.8)	42 (93.3)	0.609
Yes	3 (4.6)	6 (5.6)		1 (2.2)	3 (6.7)	
Alcoholism, *n* (%)						
No	59 (90.8)	104 (97.2)	0.1383	43 (95.6)	43 (95.6)	1
Yes	6 (9.2)	3 (2.8)		2 (4.4)	2 (4.4)	
Distance from anal verge (cm), median [IQR]	4.0 [3.0, 5.0]	4.0 [3.0, 4.8]	0.8406	4.0 [3.0, 5.0]	4.0 [3.0, 4.5]	0.9408
cT, *n* (%)						
T3	65 (100.0)	83 (77.6)	0.0001	45 (100.0)	45 (100.0)	NA
T4	0 (0.0)	24 (22.4)		0 (0.0)	0 (0.0)	
cEMVI, *n* (%)						
Negative	55(84.6)	87 (81.3)	0.5795	37 (82.2)	38 (84.4)	0.7773
Positive	10 (15.4)	20 (18.7)		8 (17.8)	7 (15.6)	
cMRF, *n* (%)						
Negative	52 (80.0)	78 (72.9)	0.2931	36 (80.0)	34 (75.6)	0.6121
Positive	13 (20.0)	29 (27.1)		9 (20.0)	11 (24.4)	
Surgery type, *n* (%)						
Dixon	22 (33.8)	20 (18.7)	0.0808	11 (24.4)	14 (31.1)	0.7777
Miles	41 (63.1)	83 (77.6)		33 (73.3)	30 (66.7)	
Hartmann	2 (3.1)	4 (3.7)		1 (2.2)	1 (2.2)	
pT, *n* (%)						
T0	0 (0.0)	22 (20.6)	< 0.0001	0 (0.0)	8 (17.8)	0.0008
T1	0 (0.0)	1 (0.9)		0 (0.0)	0 (0.0)	
T2	0 (0.0)	25 (23.4)		0 (0.0)	10 (22.2)	
T3	65 (100.0)	56 (52.3)		45 (100.0)	27 (60.0)	
T4	0 (0.0)	3 (2.8)		0 (0.0)	0 (0.0)	
pEMVI *n* (%)						
Negative	58 (89.2)	87 (81.3)	0.0433	40 (88.9)	38 (84.4)	0.2057
Positive	6 (9.2)	7 (6.5)		5 (11.1)	4 (8.9)	
Unknown	1 (1.6)	13 (12.2)		0 (0.0)	3 (6.7)	
pPNI, *n* (%)						
Negative	46 (70.8)	79 (73.8)	0.0083	33 (73.3)	35 (77.8)	0.1122
Positive	18 (27.7)	15 (14.0)		12 (26.7)	7 (15.5)	
Unknown	1 (1.5)	13 (12.2)		0 (0.0)	3 (6.7)	
CRM, *n* (%)						
Negative	63 (96.9)	104 (97.2)	1	45 (100.0)	43 (95.6)	0.4745
Positive	2 (3.1)	3 (2.8)		0 (0.0)	2 (4.4)	
Positive	2 (3.1)	3 (2.8)		0 (0.0)	2 (4.4)	

Abbreviations: ACRT, adjuvant chemoradiotherapy; cEMVI, clinical extramural vascular invasion; cMRF, clinical mesorectal fascia; CRM, circumferential resection margin; cT, clinical primary tumor category; LARC, locally advanced rectal cancer; NCRT, neoadjuvant chemoradiotherapy; pEMVI, pathological EMVI; pPNI, pathological perineural invasion; pT, pathological primary tumor category.

^a^ The *p*‐values indicate the statistical significance of differences in variable distributions between the NCRT group and ACRT group. Continuous variables were analyzed using Student's *t*‐test (normally distributed data) or the Mann–Whitney *U*‐test (non‐parametric data). Categorical variables were compared with the chi‐square test or Fisher's exact test as appropriate. All tests were two‐sided, and a *p*‐value < 0.05 was considered statistically significant.

^b^ Regarding pT, pEMVI, pPNI, and CRM, these variables were determined based on postoperative pathology. At that time, for patients who underwent NCRT, they had received chemoradiation before surgery, while patients with ACRT did not receive any chemoradiation before surgery.

**TABLE 3:** Characteristic factors of Stage III patients before and after propensity matching.

Characteristic^b^	Before propensity matching			After propensity matching		
	ACRT (*n* = 282)	NCRT (*n* = 715)	*p* ^*a*^	ACRT (*n* = 243)	NCRT (*n* = 243)	*p* ^*a*^
Gender, *n* (%)						
Male	126 (44.7)	214 (29.9)	< 0.0001	100 (41.2)	113 (46.5)	0.2726
Female	156 (55.3)	501 (70.1)		143 (58.8)	130 (53.5)	
Age (year), median [IQR]	55.5 [46.0, 61.8]	56.0 [48.0, 63.0]	0.327	55.0 [46.0, 62.0]	56.0 [48.5, 64.0]	0.3694
Smoking, *n* (%)						
No	273 (96.8)	674 (94.3)	0.1347	234 (96.3)	225 (92.6)	0.1131
Yes	9 (3.2)	41 (5.7)		9 (3.7)	18 (7.4)	
Hypertension, *n* (%)						
No	264 (93.6)	666 (93.2)	0.8992	230 (94.7)	222 (91.4)	0.2132
Yes	18 (6.4)	49 (6.8)		13 (5.3)	21 (8.6)	
Alcoholism, *n* (%)						
No	277 (98.2)	692 (96.8)	0.3031	239 (98.4)	234 (96.3)	0.2608
Yes	5 (1.8)	23 (3.2)		4 (1.6)	9 (3.7)	
Distance from anal verge (cm), median [IQR]	4.0 [3.0, 5.0]	4.0 [3.0, 5.0]	0.4463	4.0 [3.0, 5.0]	3.5 [3.0, 4.0]	0.0576
cT, *n* (%)						
T1	4 (1.4)	0 (0.0)	< 0.0001	0 (0.0)	0 (0.0)	0.8731
T2	63 (22.3)	30 (4.2)		28 (11.5)	30 (12.4)	
T3	212 (75.2)	614 (85.9)		212 (87.3)	211 (86.8)	
T4	3 (1.1)	71 (9.9)		3 (1.2)	2 (0.8)	
cN, *n* (%)						
N1	152 (53.9)	296 (41.4)	0.0005	123 (50.6)	130 (53.5)	0.5859
N2	130 (46.1)	419 (58.6)		120 (49.4)	113 (46.5)	
cEMVI, *n* (%)						
Negative	139 (49.3)	414 (57.9)	0.0137	121 (49.8)	127 (52.3)	0.5861
Positive	143 (50.7)	301 (42.1)		122 (50.2)	116 (47.7)	
cMRF, *n* (%)						
Negative	161 (57.1)	451 (62.7)	0.0804	140 (57.6)	143 (58.8)	0.7826
Positive	121 (42.9)	264 (37.3)		103 (42.4)	100 (41.2)	
Surgery type, *n* (%)						
Dixon	64 (22.7)	230 (32.2)	0.0022	51 (21.0)	75 (30.8)	0.0398
Miles	209 (74.1)	447 (62.5)		183 (75.3)	162 (66.7)	
Hartmann	9 (3.2)	38 (5.3)		9 (3.7)	6 (2.5)	
pT, *n* (%)						
T0	0 (0.0)	102 (14.3)	< 0.0001	0 (0.0)	27 (11.1)	< 0.0001
Tis	0 (0.0)	3 (0.4)		0 (0.0)	2 (0.8)	
T1	4 (1.4)	24 (3.4)		28 (11.5)	7 (2.9)	
T2	63 (22.3)	162 (22.6)		212 (87.3)	47 (19.3)	
T3	212 (75.2)	417 (58.3)		3 (1.2)	159 (65.4)	
T4	3 (1.1)	2 (0.3)		0 (0.0)	0 (0.0)	
Unknown	0 (0.0)	5 (0.7)		0 (0.0)	1 (0.4)	
pN, *n* (%)						
N0	0 (0.0)	505 (70.6)	< 0.0001	0 (0.0)	184 (75.8)	< 0.0001
N1	152 (53.9)	157 (22.0)		123 (50.6)	46 (18.9)	
N2	130 (46.1)	52 (7.3)		120 (49.4)	13 (5.3)	
Unknown	0 (0.0)	1 (0.1)		0 (0.0)	0 (0.0)	
pEMVI, *n* (%)						
Negative	140 (49.6)	630 (88.1)	< 0.0001	113 (46.5)	218 (89.7)	< 0.0001
Positive	137 (48.6)	53 (7.4)		125 (51.4)	20 (8.2)	
Unknown	5 (1.8)	32 (4.5)		5 (2.1)	5 (2.1)	
pPNI, *n* (%)						
Negative	167 (59.2)	599 (83.8)	< 0.0001	137 (56.4)	212 (87.2)	< 0.0001
Positive	112 (39.7)	86 (12.0)		103 (42.4)	27 (11.1)	
Unknown	3 (1.1)	30 (4.2)		3 (1.2)	4 (1.7)	
CRM, *n* (%)						
Negative	269 (95.4)	699 (97.8)	0.0721	230 (94.7)	236 (97.1)	0.2535
Positive	13 (4.6)	16 (2.2)		13 (5.3)	7 (2.9)	

Abbreviations: ACRT, adjuvant chemoradiotherapy; cEMVI, clinical extramural vascular invasion; cMRF, clinical mesorectal fascia; cN, clinical regional nodal category; CRM, circumferential resection margin; cT, clinical primary tumor category; LARC, locally advanced rectal cancer; NCRT, neoadjuvant chemoradiotherapy; pEMVI, pathological EMVI; pN, pathological regional nodal category; pPNI, pathological perineural invasion; pT, pathological primary tumor category.

^a^ The *p*‐values indicate the statistical significance of differences in variable distributions between the NCRT group and ACRT group. Continuous variables were analyzed using Student's *t*‐test (normally distributed data) or the Mann–Whitney *U*‐test (non‐parametric data). Categorical variables were compared with the chi‐square test or Fisher's exact test as appropriate. All tests were two‐sided, and a *p*‐value < 0.05 was considered statistically significant.

^b^ Regarding pT, pN, pEMVI, pPNI, and CRM, these variables were determined based on postoperative pathology. At that time, for patients who underwent NCRT, they had received chemoradiation before surgery, while patients with ACRT did not receive any chemoradiation before surgery.

To address the imbalanced distribution of potential confounding factors between the NCRT and ACRT groups, we conducted PSM for both Stage II and Stage III to minimize the interference of confounding factors, including the distance of the tumor from the anal verge, gender, age, clinical T stage, clinical N stage, cEMVI status, cMRF status, smoking, alcoholism, hypertension, and surgery type. After PSM, 45 paired patients in Stage II and 243 paired in Stage III were selected. There was a marked enhancement in the variable balance of the matched cohort (Tables 2 and 3). We excluded the pathological T/N stage, pEMVI, pPNI, and CRM from the PSM criteria because these variables were determined based on postoperative pathology. Given that patients in the NCRT group received treatment prior to surgery, these factors failed to accurately reflect their baseline status.

### 3.2 Oncologic Outcomes

**FIGURE 1:**
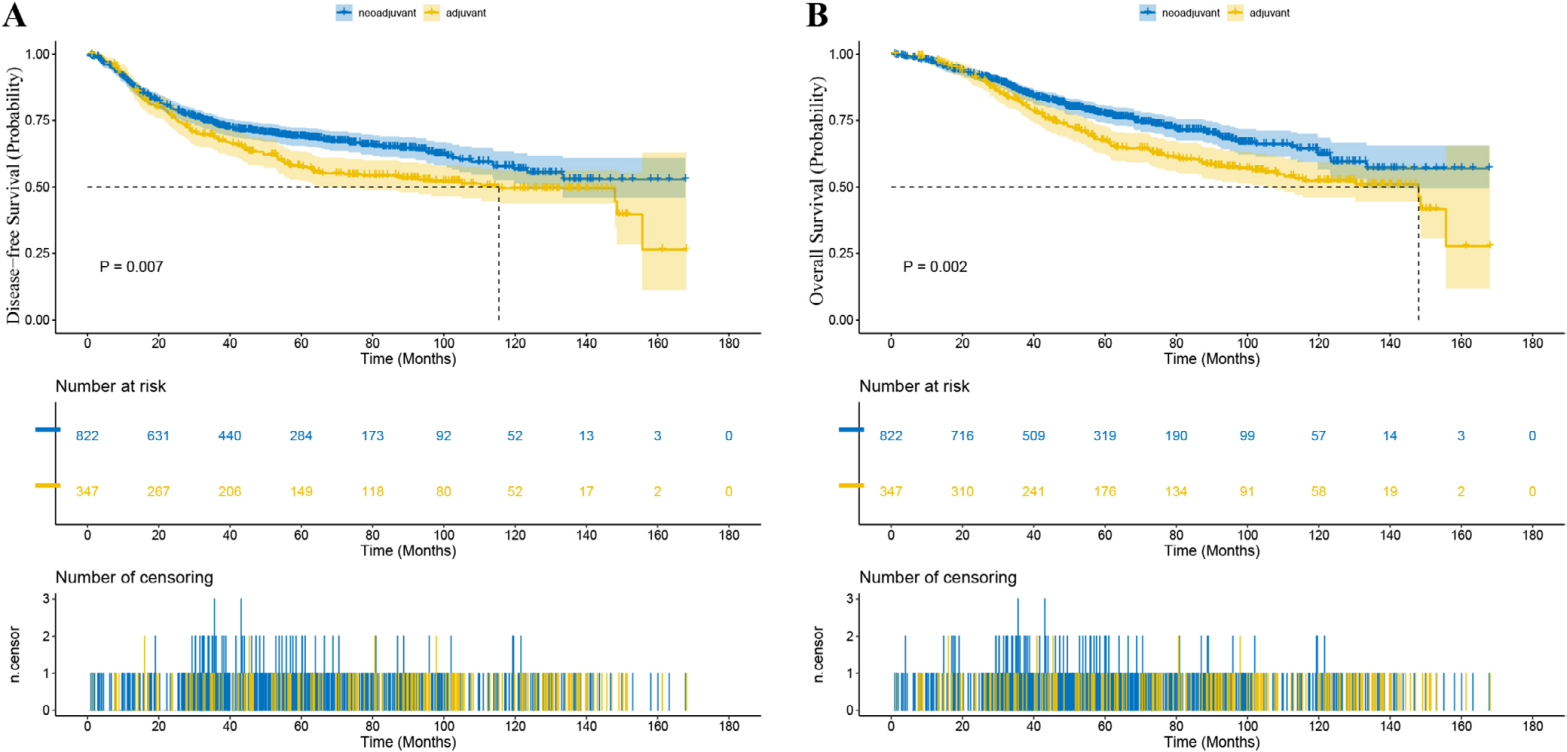
Kaplan–Meier estimates of long‐term survival in patients with LARC receiving NCRT versus ACRT: (A) DFS; (B) OS.

The study concluded on September 22, 2023. In patients with LALRC, the median length of follow‐up for the NCRT group was 49.2 months (range 0–189.9), and 58.8 months (range 0.4–175.2) for the ACRT group. The 1‐year disease‐free survival (DFS) rate was 88.74% versus 88.94% (NCRT vs. ACRT) and the 3‐year DFS rate was 73.72% versus 68.80% (*p* = 0.007, Figure 1A). The 1‐year overall survival (OS) rate was 96.91% versus 97.37% and the 3‐year OS rate was 86.07% versus 82.32% (*p* = 0.002, Figure 1B). The 1‐year local‐regional recurrence‐free survival (LRFS) rate was 95.79% versus 95.92% and the 3‐year LRFS rate was 83.96% versus 80.12% (*p* = 0.003). The 1‐year distant metastasis‐free survival (DMFS) rate was 89.48% versus 88.94% and the 3‐year DMFS rate was 74.31% versus 69.58% (*p* = 0.004).

**FIGURE 2:**
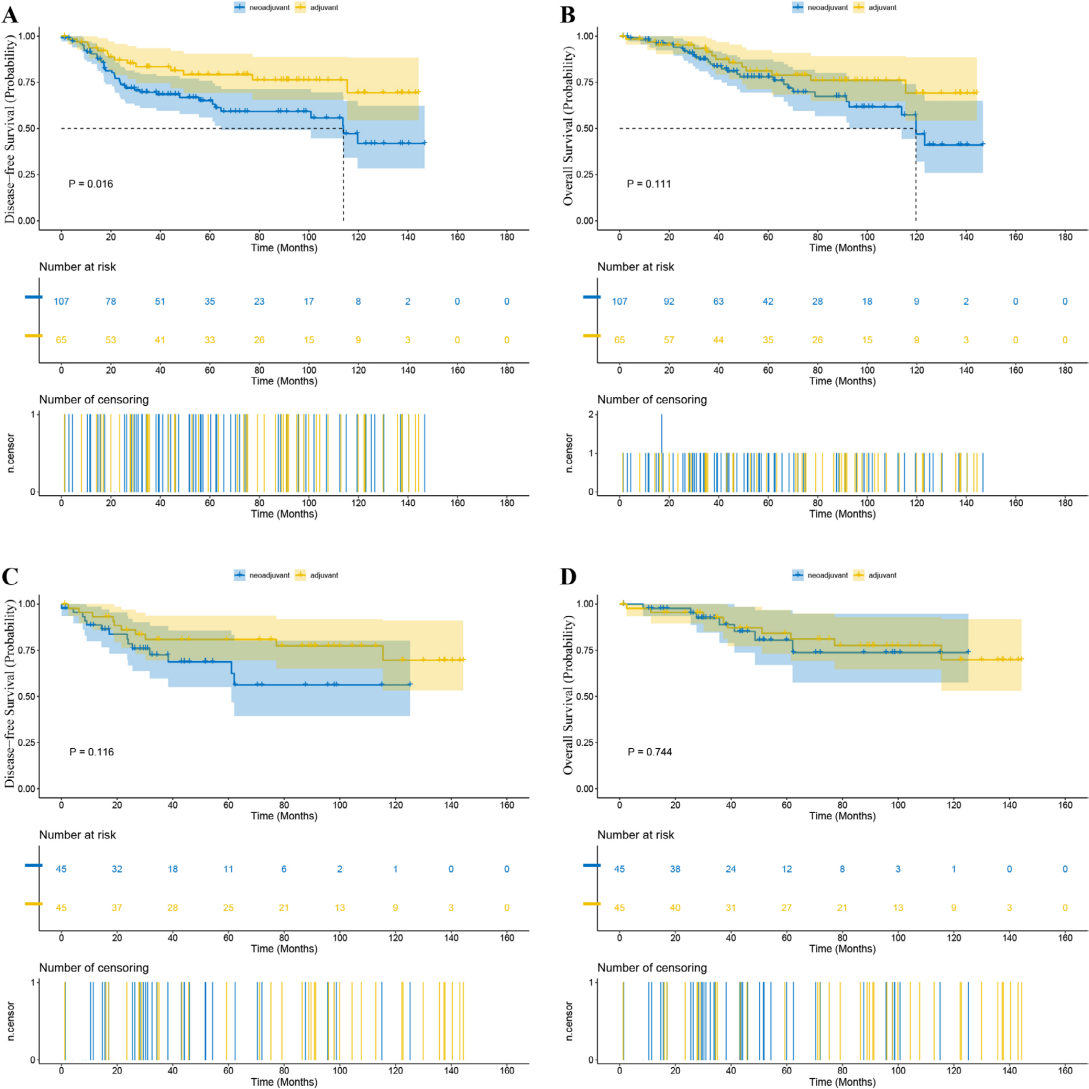
Kaplan–Meier estimates of long‐term survival in patients with Stage II LARC receiving NCRT versus ACRT: (A) DFS (before PSM); (B) OS (before PSM); (C) DFS (after PSM); (D) OS (after PSM).

**FIGURE 3:**
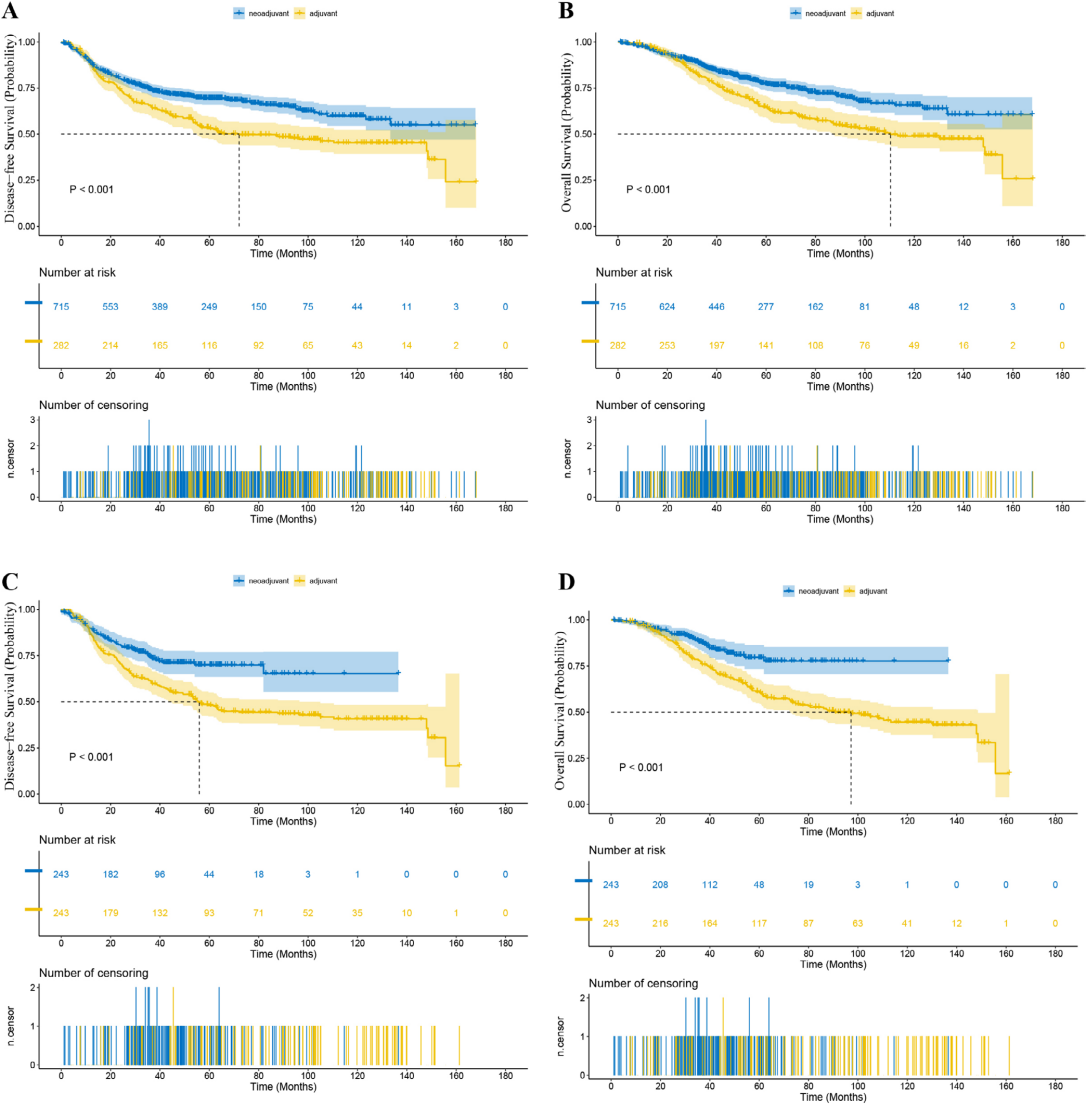
Kaplan–Meier estimates of long‐term survival in patients with Stage III LARC receiving NCRT versus ACRT: (A) DFS (before PSM); (B) OS (before PSM); (C) DFS (after PSM); (D) OS (after PSM).

Before PSM, in Stage II patients, the median length of follow‐up was 46.4 months (range 0.5–144.2) for the NCRT group and 69.9 months (range 0.4–141.8) for the ACRT group. The 1‐year DFS rate was 88.68% versus 93.18% (NCRT vs. ACRT) and the 3‐year DFS rate was 72.53% versus 80.86% (*p* = 0.016, Figure 2A). The 1‐year OS rate was 97.73% versus 95.45% and the 3‐year OS rate was 88.88% versus 92.80% (*p* = 0.111, Figure 2B). The 1‐year LRFS rate was 95.20% versus 96.85% and the 3‐year LRFS rate was 81.43% versus 91.66% (*p* = 0.069). The 1‐year DMFS rate was 91.39% versus 93.70% and the 3‐year DMFS rate was 71.52% versus 83.43% (*p* = 0.028). While in patients with Stage III tumors, the median length of follow‐up in the NCRT and ACRT groups was 49.6 months (range 0–189.9) and 58.4 months (range 4.0–175.2), respectively. The 1‐year DFS rate was 88.35% versus 87.87% (NCRT vs. ACRT) and the 3‐year DFS rate was 74.30% versus 65.63% (*p* < 0.001, Figure 3A). The 1‐year OS rate was 96.73% versus 97.49% and the 3‐year OS rate was 86.05% versus 80.35% (*p* < 0.001, Figure 3B). The 1‐year LRFS rate was 95.88% versus 95.71% and the 3‐year LRFS rate was 84.33% versus 77.63% (*p* < 0.001). The 1‐year DMFS rate was 89.20% versus 87.87% and the 3‐year DMFS rate was 74.71% versus 66.58% (*p* < 0.001).

After PSM, in Stage II patients, the median length of follow‐up was 42.3 months (range 0.7–123.0) for the NCRT group and 75.7 months (range 0.4–141.8) for the ACRT group. The 1‐year DFS rate was 88.68% versus 93.18% (NCRT vs. ACRT), and the 3‐year DFS rate was 72.53% versus 80.86% (*p* = 0.116, Figure 2C). The 1‐year OS rate was 97.73% versus 95.45%, and the 3‐year OS rate was 88.88% versus 92.80% (*p* = 0.744, Figure 2D). The 1‐year LRFS rate was 95.45% vs. 95.45%, and the 3‐year LRFS rate was 81.74% versus 93.07% (*p* = 0.433). The 1‐year DMFS rate was 90.96% versus 93.18%, and the 3‐year DMFS rate was 77.20% versus 80.86% (*p* = 0.262). And in terms of the risk of death or recurrence, there wasn't a significant difference between the NCRT group and the ACRT group (OS: hazard ratio (HR) = 1.18, 95% confidence interval (CI) 0.43–3.27, *p* = 0.7445; DFS: HR = 1.93, CI 0.84–4.46, *p* = 0.1217). While in matched Stage III patients, the median length of follow‐up was 37.6 months (range 0–134.3) for the NCRT group and 55.8 months (range 4.1–158.6) for the ACRT group. The 1‐year DFS rate was 89.89% versus 86.76% (NCRT vs. ACRT), and the 3‐year DFS rate was 74.68% versus 61.53% (*p* < 0.001, Figure 3C). The 1‐year OS rate was 97.88% versus 97.09%, and the 3‐year OS rate was 88.09% versus 77.69% (*p* < 0.001, Figure 3D). The 1‐year LRFS rate was 97.47% versus 95.43%, and the 3‐year LRFS rate was 86.36% versus 74.98% (*p* < 0.001). The 1‐year DMFS rate was 90.31% versus 86.76%, and the 3‐year DMFS rate was 75.99% versus 62.19% (*p* < 0.001). Besides, NCRT was associated with a significant reduction in the risk of death and recurrence versus ACRT (OS: HR = 0.47, 95% CI 0.32–0.69, *p* = 0.0001; DFS: HR = 0.55, CI 0.40–0.74, *p* = 0.0001).

## 4 Discussion

NCRT followed by TME has become the standard treatment for LARC, as it has been shown to reduce local recurrence rates more effectively than ACRT [13, 14, 15, 16, 19]. However, the debate over whether NCRT can improve long‐term survival for patients with LARC continues.

In our research, we found, after PSM, for patients with Stage III LALRC, NCRT significantly increased the 3‐year DFS rate by 13.15% and the 3‐year OS rate by 10.40%, in comparison to ACRT. The observed improvement in OS with NCRT may be explained by several mechanisms. First, while NCRT is being delivered, the intact primary tumor allows radiotherapy and chemotherapy to induce immunogenic cell death (ICD) [23, 24]. Radiotherapy in particular can serve as an ‘in situ vaccination’, releasing tumor‐associated antigens and damage‐associated molecular patterns (DAMPs) that prime systemic anti‐tumor immunity [25, 26, 27]. This immunostimulatory effect facilitates the eradication of micrometastatic disease and establishes durable immune surveillance through circulating immuner cells and immune cells residing in tumor draining lymph nodes, thereby lowering the risk of local recurrence and distant metastasis [28, 29]. These immunologic considerations are consistent with our findings that Stage III patients treated with NCRT exhibited superior LRFS, DMFS, and DFS compared with those receiving ACRT. Because distant metastasis is a principal determinant of OS, NCRT‐mediated suppression of distant metastasis directly contributes to the observed overall survival advantage. In contrast, ACRT is administered after resection of the primary tumor; consequently, its capacity to induce ICD and activate systemic immune surveillance is markedly diminished. In addition, radiotherapy efficacy is highly dependent on tissue [30, 31]. The postoperative tumor bed is relatively hypoxic, owing to disrupted vasculature and the formation of hypoxic niches, rendering residual tumor cells more radio‐resistant than the better‐oxygenated primary tumor exposed to preoperative irradiation. This hypoxia‐mediated radio‐resistance likely underlies the inferior local and distant control achieved with ACRT, and ultimately, its poorer OS outcomes.

Importantly, our retrospective analysis controlled for chemotherapy intensity: both the NCRT and ACRT cohorts received at least six cycles of chemotherapy. The OS benefit associated with NCRT therefore cannot be ascribed to greater chemotherapy dose density. However, clinical experience indicates that patients undergoing ACRT often exhibit lower treatment tolerance, leading to early discontinuation or dose reduction, which may further compromise overall survival relative to NCRT.

Whereas, the NSABP R‐03 trial reported a 5‐year overall survival (OS) for preoperative patients at 74.5% versus 65.6% for postoperative patients (*p* = 0.065) [14]. The German CAO/ARO/AIO‐94 trial found no significant difference in 5‐year DFS/OS or 10‐year DFS/OS between preoperative patients and postoperative patients [16]. Similarly, the MRC CR07 trial showed a 3‐year OS for preoperative patients at 80.3% versus 78.6% for postoperative patients (*p* = 0.40) [15]. These three phase III randomized clinical trials demonstrated no significant difference in OS between NCRT and ACRT, which seemed to conflict with the results from our retrospective study, although the NSABP R‐03 trial had already demonstrated a trend consistent with our findings.

We hypothesized several reasons for our findings that NCRT significantly benefited the survival of patients with Stage III LALRC: (1) The clinical trials focused on patients with LARC without further distinguishing between Stage II and Stage III. Our results suggested that Stage III patients benefit significantly from NCRT, while Stage II patients do not, indicating potential confounding factors in these clinical studies. A retrospective study with a smaller sample size focused on Stage III patients and also found that NCRT improved the OS and DFS of Stage III patients, consistent with our results [32]; (2) We focused solely on patients with LALRC, whereas the three phase III RCTs mentioned above did not further categorize patients by the distance of the tumor from the anal verge. Many studies have indicated that the distance of the tumor from the anal verge had a profound impact on the efficacy of NCRT [33, 34, 35]. We speculated that patients with LALRC were more likely to have long‐term survival benefited from NCRT compared to patients with middle or high rectal cancer, and hence tumor location was another potential confounding factor not addressed in these Phase III clinical studies.

It's noteworthy that our study found no improvement in OS or DFS for Stage II patients when comparing NCRT to ACRT. We hypothesized that this might be due to: (1) Stage II patients had less severe conditions, so the treatment intensity of ACRT was strong enough to achieve similar long‐term survival results as NCRT did; (2) Stage II patients, characterized by smaller tumor lesions, limited infiltration, and no lymph node metastasis, would have a fairly low likelihood of recurrence or metastasis after TME surgery, even though without the help of NCRT to shrink the tumor, which led to a good long‐term survival result for patients receiving either NCRT or ACRT.

Besides the definite reduction of local recurrence rate and the possible benefits of long‐term survival rate, NCRT offered numerous merits unattainable by ACRT [36]. According to the NSABP R‐03 trial, 15% of patients receiving NCRT could achieve pCR and hence obtain an exceptional 5‐year OS rate of 87.8% [14]. With the introduction of total neoadjuvant therapy (TNT), namely preoperative chemoradiotherapy plus induction or consolidation chemotherapy, the pCR rate has been elevated significantly. Based on the findings of the CAO/ARO/AIO‐12 randomized clinical trial, the pCR rate of patients with LARC receiving TNT with consolidation chemotherapy has increased to 25% [37]. An analysis combining individual patient data revealed that patients with pCR following chemoradiation had superior long‐term outcomes compared to those without pCR, as indicated by the 5‐year crude DFS rate of 83.3% compared to 65.6% [38].

Compared with the prevailing corpus of research scrutinizing the protracted survival outcomes in LARC patients undergoing NCRT and ACRT, our investigation distinguished itself by the rigorous segmentation of the LARC demographic, with an intensified focus on Stages II and III of low rectal cancer. This approach had culminated in the assembly of a significant cohort for Stage III low rectal cancer, bolstered by an extensive follow‐up duration. Furthermore, our adoption of PSM had been instrumental in neutralizing underlying confounders, thereby enhancing the credibility of our findings.

However, our study had several constraints. Initially, it was retrospective and conducted at a single center. Besides, we failed to include a substantial cohort for Stage II low rectal cancer, and the statistical strength could have decreased due to the small sample size. Additionally, the variables accounted for in our baseline statistics were somewhat limited, and despite the application of PSM, there remained the possibility of unaccounted confounding factors. What's more, our study did not assess patient‐reported quality of life (QoL) or late treatment‐related toxicities. This represents an important limitation, given that late‐toxicity profiles may differ substantially between NCRT and ACRT, with ACRT generally associated with a higher incidence of delayed adverse events.

## Author Contributions

Conceptualization: Siyuan Chen, Ruiyan Wu, Juefeng Wan, Ye Xu, Zhen Zhang, Hongtu Zheng. Methodology: Siyuan Chen, Ruiyan Wu, Juefeng Wan, Yaqi Wang, Zhiyuan Zhang, Ye Xu, Zhen Zhang, Hongtu Zheng. Software: Siyuan Chen. Data curation: Siyuan Chen, Yun Xu, Hongtu Zheng. Investigation: Siyuan Chen, Ruiyan Wu, Juefeng Wan, Yun Xu, Yaqi Wang, Zhiyuan Zhang, Lili Huang, Yujun Liu, Yingxuan Lin, Luoxi He, Yun Deng, Fan Xia, Ye Xu, Zhen Zhang, Hongtu Zheng. Validation: Siyuan Chen, Hongtu Zheng. Formal analysis: Siyuan Chen, Hongtu Zheng. Supervision: Ruiyan Wu, Juefeng Wan, Fan Xia, Ye Xu, Zhen Zhang, Hongtu Zheng. Funding acquisition: Ruiyan Wu, Juefeng Wan, Fan Xia, Ye Xu, Hongtu Zheng. Visualization: Siyuan Chen, Hongtu Zheng. Project administration: Juefeng Wan, Ye Xu, Zhen Zhang, Hongtu Zheng. Resources: Ruiyan Wu, Juefeng Wan, Yun Xu, Fan Xia, Ye Xu, Zhen Zhang, Hongtu Zheng. Writing – original draft: Siyuan Chen. Writing – review and editing: Siyuan Chen, Ruiyan Wu, Juefeng Wan, Yun Xu, Yaqi Wang, Zhiyuan Zhang, Fan Xia, Ye Xu, Zhen Zhang, Hongtu Zheng.

## Ethics Statement

The study's ethical code, 2111246‐26, was approved by the Fudan University Shanghai Cancer Center's ethics committee on November 22, 2021. Every patient gave their informed permission to participate and the publishing of all data included in the manuscript.

## Conflicts of Interest

The authors declare no conflicts of interest.

## Data Availability

Research data are stored in the repository of Fudan University Shanghai Cancer Center and will be shared upon request to the corresponding author.

## References

[1] H. Sung , J. Ferlay , R. L. Siegel , et al., “Global Cancer Statistics 2020: Globocan Estimates of Incidence and Mortality Worldwide for 36 Cancers in 185 Countries,” CA: A Cancer Journal for Clinicians 71 (2021): 209–249. 33538338 10.3322/caac.21660

[2] S. Murugappan , W. P. Harris , C. G. Willett , and E. Lin , “Multidisciplinary Management of Locally Advanced Rectal Cancer: Neoadjuvant Approaches,” Journal of the National Comprehensive Cancer Network 11 (2013): 548–557. 23667205 10.6004/jnccn.2013.0071

[3] R. Qu , Y. Ma , L. Tao , et al., “Features of Colorectal Cancer in China Stratified by Anatomic Sites: A Hospital‐Based Study Conducted in University‐Affiliated Hospitals From 2014 to 2018,” Chinese Journal of Cancer Research 33 (2021): 500–511. 34584375 10.21147/j.issn.1000-9604.2021.04.07 PMC8435820

[4] C. E. Bailey , C. Y. Hu , Y. N. You , et al., “Increasing Disparities in the Age‐Related Incidences of Colon and Rectal Cancers in the United States, 1975‐2010,” JAMA Surgery 150 (2015): 17–22. 25372703 10.1001/jamasurg.2014.1756 PMC4666003

[5] J. Zhu , A. Liu , X. Sun , et al., “Multicenter, Randomized, Phase Iii Trial of Neoadjuvant Chemoradiation With Capecitabine and Irinotecan Guided by ugt1a1 Status in Patients With Locally Advanced Rectal Cancer,” Journal of Clinical Oncology 38 (2020): 4231–4239. 33119477 10.1200/JCO.20.01932 PMC7768334

[6] M. Aklilu and C. Eng , “The Current Landscape of Locally Advanced Rectal Cancer,” Nature Reviews. Clinical Oncology 8 (2011): 649–659. 10.1038/nrclinonc.2011.118 21826084

[7] S. X. Roodbeen , A. Spinelli , W. A. Bemelman , et al., “Local Recurrence After Transanal Total Mesorectal Excision for Rectal Cancer: A Multicenter Cohort Study,” Annals of Surgery 274 (2021): 359–366. 31972648 10.1097/SLA.0000000000003757

[8] S. J. Bains , H. Abrahamsson , K. Flatmark , et al., “Immunogenic Cell Death by Neoadjuvant Oxaliplatin and Radiation Protects Against Metastatic Failure in High‐Risk Rectal Cancer,” Cancer Immunology, Immunotherapy 69 (2020): 355–364. 31893287 10.1007/s00262-019-02458-x PMC7044156

[9] J. J. Smith and J. Garcia‐Aguilar , “Advances and Challenges in Treatment of Locally Advanced Rectal Cancer,” Journal of Clinical Oncology 33 (2015): 1797–1808. 25918296 10.1200/JCO.2014.60.1054 PMC4559608

[10] R. J. Heald and R. D. Ryall , “Recurrence and Survival After Total Mesorectal Excision for Rectal Cancer,” Lancet 1 (1986): 1479–1482. 2425199 10.1016/s0140-6736(86)91510-2

[11] Gastrointestinal Tumor Study Group , “Prolongation of the Disease‐Free Interval in Surgically Treated Rectal Carcinoma,” New England Journal of Medicine 312 (1985): 1465–1472. 2859523 10.1056/NEJM198506063122301

[12] J. E. Krook , C. G. Moertel , L. L. Gunderson , et al., “Effective Surgical Adjuvant Therapy for High‐Risk Rectal Carcinoma,” New England Journal of Medicine 324 (1991): 709–715. 1997835 10.1056/NEJM199103143241101

[13] R. Sauer , H. Becker , W. Hohenberger , et al., “Preoperative Versus Postoperative Chemoradiotherapy for Rectal Cancer,” New England Journal of Medicine 351 (2004): 1731–1740. 15496622 10.1056/NEJMoa040694

[14] M. S. Roh , L. H. Colangelo , M. J. O'Connell , et al., “Preoperative Multimodality Therapy Improves Disease‐Free Survival in Patients With Carcinoma of the Rectum: Nsabp r‐03,” Journal of Clinical Oncology 27 (2009): 5124–5130. 19770376 10.1200/JCO.2009.22.0467 PMC2773471

[15] P. Quirke , R. Steele , J. Monson , et al., “Effect of the Plane of Surgery Achieved on Local Recurrence in Patients With Operable Rectal Cancer: A Prospective Study Using Data From the Mrc cr07 and Ncic‐Ctg co16 Randomised Clinical Trial,” Lancet 373 (2009): 821–828. 19269520 10.1016/S0140-6736(09)60485-2 PMC2668948

[16] R. Sauer , T. Liersch , S. Merkel , et al., “Preoperative Versus Postoperative Chemoradiotherapy for Locally Advanced Rectal Cancer: Results of the German Cao/Aro/Aio‐94 Randomized Phase Iii Trial After a Median Follow‐Up of 11 Years,” Journal of Clinical Oncology 30 (2012): 1926–1933. 22529255 10.1200/JCO.2011.40.1836

[17] F. Dossa , T. R. Chesney , S. A. Acuna , and N. N. Baxter , “A Watch‐And‐Wait Approach for Locally Advanced Rectal Cancer After a Clinical Complete Response Following Neoadjuvant Chemoradiation: A Systematic Review and Meta‐Analysis,” Lancet Gastroenterology & Hepatology 2 (2017): 501–513. 28479372 10.1016/S2468-1253(17)30074-2

[18] A. G. Renehan , L. Malcomson , R. Emsley , et al., “Watch‐and‐Wait Approach Versus Surgical Resection After Chemoradiotherapy for Patients With Rectal Cancer (The Oncore Project): A Propensity‐Score Matched Cohort Analysis,” Lancet Oncology 17 (2016): 174–183. 26705854 10.1016/S1470-2045(15)00467-2

[19] A. B. Benson , A. P. Venook , M. M. Al‐Hawary , et al., “Colon Cancer, Version 2.2021, Nccn Clinical Practice Guidelines in Oncology,” Journal of the National Comprehensive Cancer Network 19 (2021): 329–359. 33724754 10.6004/jnccn.2021.0012

[20] M. Frasson , E. Garcia‐Granero , D. Roda , et al., “Preoperative Chemoradiation May Not Always Be Needed for Patients With t3 and t2n+ Rectal Cancer,” Cancer 117 (2011): 3118–3125. 21264832 10.1002/cncr.25866

[21] D. Schrag , Q. Shi , M. R. Weiser , et al., “Preoperative Treatment of Locally Advanced Rectal Cancer,” New England Journal of Medicine 389 (2023): 322–334. 37272534 10.1056/NEJMoa2303269 PMC10775881

[22] M. R. Weiser , “Ajcc 8th Edition: Colorectal Cancer,” Annals of Surgical Oncology 25 (2018): 1454–1455. 29616422 10.1245/s10434-018-6462-1

[23] J. Sprooten , R. S. Laureano , I. Vanmeerbeek , et al., “Trial Watch: Chemotherapy‐Induced Immunogenic Cell Death in Oncology,” Oncoimmunology 12 (2023): 2219591. 37284695 10.1080/2162402X.2023.2219591 PMC10240992

[24] S. Zhu , Y. Wang , J. Tang , and M. Cao , “Radiotherapy Induced Immunogenic Cell Death by Remodeling Tumor Immune Microenvironment,” Frontiers in Immunology 13 (2022): 1074477. 36532071 10.3389/fimmu.2022.1074477 PMC9753984

[25] Y. Wang , Y. Li , Y. Yang , et al., “In Situ Vaccination Caused by Diverse Irradiation‐Driven Cell Death Programs,” Theranostics 14 (2024): 1147–1167. 38323315 10.7150/thno.86004 PMC10845208

[26] P. M. Carlson , R. B. Patel , J. Birstler , et al., “Radiation to All Macroscopic Sites of Tumor Permits Greater Systemic Antitumor Response to in Situ Vaccination,” Journal for Immunotherapy of Cancer 11 (2023): e005463. 36639155 10.1136/jitc-2022-005463 PMC9843201

[27] S. Yasmin‐Karim , J. Wood , J. Wirtz , et al., “Optimizing In Situ Vaccination During Radiotherapy,” Frontiers in Oncology 11 (2021): 711078. 34765538 10.3389/fonc.2021.711078 PMC8577814

[28] C. L. Wang , A. S. Ho , C. C. Chang , et al., “Radiotherapy Enhances CXCR3^high^CD8^+^ T Cell Activation Through Inducing IFNγ‐Mediated CXCL10 and ICAM‐1 Expression in Lung Cancer Cells,” Cancer Immunology, Immunotherapy 72 (2023): 1865–1880. 36688994 10.1007/s00262-023-03379-6 PMC10198930

[29] L. Sloan , R. Sen , C. Liu , et al., “Radiation Immunodynamics in Patients With Glioblastoma Receiving Chemoradiation,” Frontiers in Immunology 15 (2024): 1438044. 39346903 10.3389/fimmu.2024.1438044 PMC11427284

[30] A. Rakotomalala , A. Escande , A. Furlan , S. Meignan , and E. Lartigau , “Hypoxia in Solid Tumors: How Low Oxygenation Impacts the “Six Rs” of Radiotherapy,” Frontiers in Endocrinology 12 (2021): 742215. 34539584 10.3389/fendo.2021.742215 PMC8445158

[31] M. H. Bennett , J. Feldmeier , R. Smee , and C. Milross , “Hyperbaric Oxygenation for Tumour Sensitisation to Radiotherapy,” Cochrane Database of Systematic Reviews 4 (2018): CD005007. 16235387 10.1002/14651858.CD005007.pub2

[32] J. Kang , S. M. Jang , J. H. Baek , W. S. Lee , and T. H. Cho , “Short‐Term Results and Long‐Term Oncologic Outcomes Between Neoadjuvant Chemoradiotherapy and Adjuvant Postoperative Chemoradiotherapy for Stage III Rectal Cancer: A Case‐Matched Study,” Annals of Surgical Oncology 19 (2012): 2494–2499. 22476817 10.1245/s10434-012-2311-9

[33] A. Restivo , L. Zorcolo , I. M. Cocco , et al., “Elevated Cea Levels and Low Distance of the Tumor From the Anal Verge Are Predictors of Incomplete Response to Chemoradiation in Patients With Rectal Cancer,” Annals of Surgical Oncology 20 (2013): 864–871. 23010737 10.1245/s10434-012-2669-8

[34] W. van Gijn , C. A. Marijnen , I. D. Nagtegaal , et al., “Preoperative Radiotherapy Combined With Total Mesorectal Excision for Resectable Rectal Cancer: 12‐Year Follow‐Up of the Multicentre, Randomised Controlled Tme Trial,” Lancet Oncology 12 (2011): 575–582. 21596621 10.1016/S1470-2045(11)70097-3

[35] M. D. Santos , C. Silva , A. Rocha , et al., “Predictive Clinical Model of Tumor Response After Chemoradiation in Rectal Cancer,” Oncotarget 8 (2017): 58133–58151. 28938543 10.18632/oncotarget.19651 PMC5601639

[36] A. Hartley , K. F. Ho , C. McConkey , and J. I. Geh , “Pathological Complete Response Following Pre‐Operative Chemoradiotherapy in Rectal Cancer: Analysis of Phase II/III Trials,” British Journal of Radiology 78 (2005): 934–938. 16177017 10.1259/bjr/86650067

[37] E. Fokas , M. Allgäuer , B. Polat , et al., “Randomized Phase Ii Trial of Chemoradiotherapy Plus Induction or Consolidation Chemotherapy as Total Neoadjuvant Therapy for Locally Advanced Rectal Cancer: Cao/Aro/Aio‐12,” Journal of Clinical Oncology 37 (2019): 3212–3222. 31150315 10.1200/JCO.19.00308

[38] M. Maas , P. J. Nelemans , V. Valentini , et al., “Long‐Term Outcome in Patients With a Pathological Complete Response After Chemoradiation for Rectal Cancer: A Pooled Analysis of Individual Patient Data,” Lancet Oncology 11 (2010): 835–844. 20692872 10.1016/S1470-2045(10)70172-8

